# Mesenchymal stromal cells inhibit NLRP3 inflammasome activation in a model of Coxsackievirus B3-induced inflammatory cardiomyopathy

**DOI:** 10.1038/s41598-018-20686-6

**Published:** 2018-02-12

**Authors:** Kapka Miteva, Kathleen Pappritz, Marzena Sosnowski, Muhammad El-Shafeey, Irene Müller, Fengquan Dong, Konstantinos Savvatis, Jochen Ringe, Carsten Tschöpe, Sophie Van Linthout

**Affiliations:** 1 Berlin-Brandenburg Center for Regenerative Therapies, Charité, University Medicine Berlin, Campus Virchow, Berlin, Germany; 2 DZHK (German Center for Cardiovascular Research), partner site Berlin, Berlin, Germany; 3 Medical Biotechnology Research Department, Genetic Engineering and Biotechnology Research Institute (GEBRI), City of Scientific Research and Technological Applications, Alexandria, Egypt; 4 Laboratory for Tissue Engineering, Charité, University Medicine Berlin, Berlin, Germany; 5 Charité-University-Medicine Berlin, Campus Rudolf Virchow, Department of Cardiology, Berlin, Germany

## Abstract

Inflammation in myocarditis induces cardiac injury and triggers disease progression to heart failure. NLRP3 inflammasome activation is a newly identified amplifying step in the pathogenesis of myocarditis. We previously have demonstrated that mesenchymal stromal cells (MSC) are cardioprotective in Coxsackievirus B3 (CVB3)-induced myocarditis. In this study, MSC markedly inhibited left ventricular (LV) NOD2, NLRP3, ASC, caspase-1, IL-1β, and IL-18 mRNA expression in CVB3-infected mice. ASC protein expression, essential for NLRP3 inflammasome assembly, increased upon CVB3 infection and was abrogated in MSC-treated mice. Concomitantly, CVB3 infection *in vitro* induced NOD2 expression, NLRP3 inflammasome activation and IL-1β secretion in HL-1 cells, which was abolished after MSC supplementation. The inhibitory effect of MSC on NLRP3 inflammasome activity in HL-1 cells was partly mediated via secretion of the anti-oxidative protein stanniocalcin-1. Furthermore, MSC application in CVB3-infected mice reduced the percentage of NOD2-, ASC-, p10- and/or IL-1β-positive splenic macrophages, natural killer cells, and dendritic cells. The suppressive effect of MSC on inflammasome activation was associated with normalized expression of prominent regulators of myocardial contractility and fibrosis to levels comparable to control mice. In conclusion, MSC treatment in myocarditis could be a promising strategy limiting the adverse consequences of cardiac and systemic NLRP3 inflammasome activation.

## Introduction

Coxsackievirus B3 (CVB3) infection is a predominant cause of viral myocarditis, which can induce sudden death in young adults or progress to dilated cardiomyopathy. Main characteristics of viral myocarditis are a local and systemic inflammatory response. While inflammatory processes are a necessary immune defense for ultimate viral elimination and healing, inflammation induces cardiac injury and represent a major mechanism in the pathogenesis of myocarditis^1^. The innate immunity is the first line of defense, which senses via pattern-recognition receptors (PRRs) signals of “danger,” including pathogen-associated molecular patterns (PAMPs) and host-derived signals of cellular stress, known as danger-associated molecular patterns (DAMPs)^2,3^. One of the four main classes of PRRs are nucleotide-binding oligomerization domain (NOD)-like receptors (NLRs), which are intracellular receptors and can be assembled into larger structures, termed inflammasomes^4^. NOD2 is a cytoplasmatic PRR member of the NLR family, which recognizes CVB3^5^, plays an important role in the innate immune response and is associated with cardiac injury by exacerbation of inflammation and cardiac remodeling^6^. NOD2 activates (NOD)-like receptor pyrin domain-containing 3 (NLRP3) inflammasome and IL-1β processing and secretion^4^, primarily on phagocytic antigen-presenting cells such as macrophages and dendritic cells (DCs). Activated NLRP3 interacts with the adapter apoptosis-associated speck-like protein containing a C-terminal caspase recruitment domain (ASC), resulting in recruitment of pro-caspase-1 and induction of auto-cleavage of caspase-1^4^. Caspase-1 is known as the inflammatory caspase essential in the maturation of the two highly pro-inflammatory interleukins (IL)−1 family cytokines: IL-1β and IL-18, known to exacerbate the inflammatory responses and induce cardio-depressive effects and cardiac remodeling^7–9^. The NLRP3 inflammasome has been identified as a central player in the development of heart diseases. NLRP3 inflammasome activation in resident cardiomyocytes and fibroblasts leads to myocardial dysfunction, while inflammasome inhibition limits inflammation and myocardial fibrosis^10,11^.

CVB3 infection induces NLRP3 inflammasome activation *in vitro* and *in vivo*, which inhibition has been shown to significantly alleviate the severity of CVB3-induced myocarditis and to improve cardiac function^12^. The clinical relevance of NOD2 and NLRP3 in CVB3-induced myocarditis follows from recent own findings in CVB3-postive myocarditis patients which show that cardiac NOD2 and NLRP3 expression drops in CVB3-positive patients who eliminated the virus and improved cardiac function over time^13^. Therefore, inhibition of NLRP3 inflammasome activation or IL-1β may be a valuable therapeutic strategy to limit the inflammatory response, which high intensity is a clear negative prognostic marker^14^.

Mesenchymal stromal cells (MSC) are effective immunomodulators of the immune responses^15–17^. We have demonstrated that intravenous (i.v.) MSC application has a cardioprotective effect in CVB3-induced inflammatory cardiomyopathy since MSC cannot be infected with CVB3, exert anti-viral effects, reduce CVB3-associated cardiomyocyte apoptosis, myocardial fibrosis, inflammation, improve left ventricle (LV) function and induce prominent systemic immunomodulation^18,19^.

Taking into account that the NLRP3 inflammasome is known to play a crucial role in the pathogenesis of CVB3-induced myocarditis and MSC have been shown to inhibit NLRP3 inflammasome activation in macrophages^20^, the aim of the present study was to evaluate the effect of i.v. MSC application on NLRP3 inflammasome activation in murine CVB3-induced myocarditis. We could demonstrate that MSC potently inhibit the NLRP3 inflammasome activation in the heart and mediate systemic immunoregulation via abrogating NLRP3 inflammasome activation and IL-1β secretion in cells of the innate immune system, which could interrupt the inflammatory process amplification and subsequently diminish the adverse cardiac inflammation and dysfunction following myocardial injury.

## Results

### Mesenchymal stromal cells inhibit NOD2 expression and NLRP3 inflammasome activation in the heart of Coxsackievirus B3-infected mice

**Figure 1:**
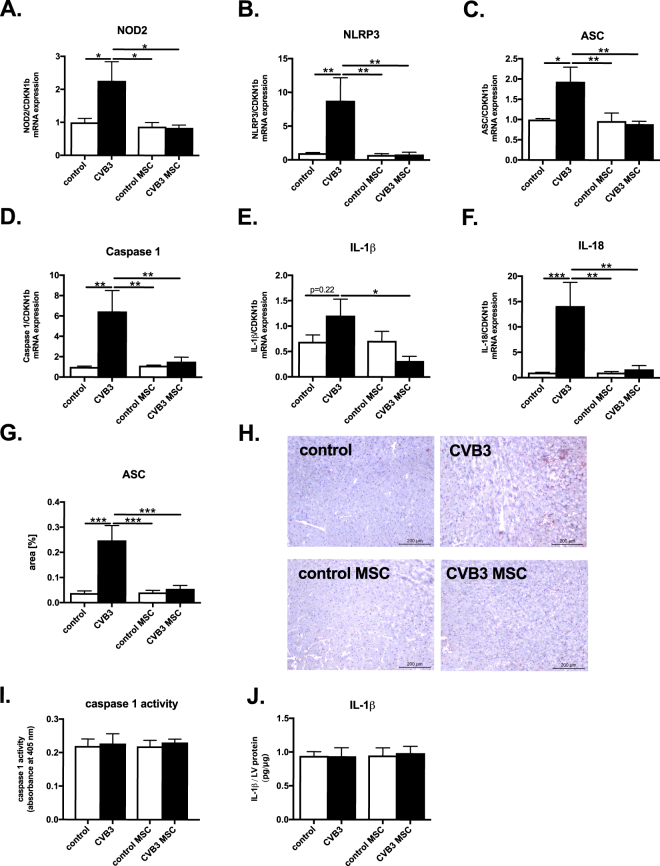
Mesenchymal stromal cells abrogate Coxsackievirus B3-induced expression of NOD2 and NLRP3 inflammasome components and products in the heart. Bar graphs represent the mean ± SEM of (**A**) NOD2, (**B**) NLRP3, (**C**) ASC, (**D**) Caspase 1, (**E**) IL-1β and (**F**) IL-18 mRNA expression in the LV of control, CVB3, control MSC, and CVB3 MSC mice, as indicated, with n = 5–6/group and *p < 0.05 and **p < 0.01. (**G**) Bar graphs represent the mean ± SEM of a quantitative analysis ASC positive area in the heart of control, CVB3, control MSC, and CVB3 MSC mice, as indicated, with n = 4–6/group and ***p < 0.001. (**H**) ASC expression in LV of control mice receiving PBS (upper left panel) or MSC (lower left panel), or of CVB3 infected mice receiving PBS (upper right panel) or MSC (lower right panel), detected by immunohistochemistry at a magnification of ×100. Bar graphs represent the mean ± SEM of (**I**) LV caspase 1 activity represented as absorbance at 405 nm and (**J**) IL-1β protein expression, depicted as pg IL-1ß per µg LV protein, determined in the LV of control, CVB3, control MSC, and CVB3 MSC mice, with n = 5–6/group.

To investigate the effects of MSC on NLRP3 inflammasome activation, one million MSC were i.v. injected one day post CVB3 infection in C57BL/6 mice. CVB3 infection induced prominent NOD2 expression and NLRP3 inflammasome activation in the heart, as indicated by a 2.3-fold (p < 0.05) and 8.8-fold (p < 0.005) increase in LV NOD2 and NLRP3 mRNA expression, respectively. In parallel, CVB3 mice exhibited a 1.9-fold (p < 0.05), 6.5-fold (p < 0.005), 1.7-fold (p = 0.22), and 14.0-fold (p < 0.001) upregulated LV expression of ASC, caspase 1, IL-1β and IL-18, respectively (Fig. 1A–F). Importantly, MSC application in CVB3-infected mice not only reduced the LV expression of NOD2 by 2.7-fold (p < 0.05) and NLRP3 by 10.6–fold (p < 0.005), but also of ASC, caspase 1, IL-1β, and IL-18 by 2.1-fold (p < 0.01), 4.3-fold (p < 0.005), 3.8-fold (p < 0.05) and 8.2-fold (p < 0.005), respectively versus CVB3 mice. Furthermore, immunohistochemical analysis showed a significant 6.6-fold (p < 0.001) upregulation in ASC expression in the heart of CVB3-infected mice versus control mice (Fig. 1G and H). MSC treatment of CVB3 mice prominently downregulated the LV ASC protein expression by 4.5-fold (p < 0.001) versus CVB3 mice. NLRP3 inflammasome components downstream of ASC remained unchanged as shown by unaltered LV caspase 1 activity (Fig. 1I), and IL-1ß protein expression (Fig. 1J) in CVB3-infected compared to control mice and they remained unaffected by MSC application.

### Mesenchymal stromal cells suppress NOD2 expression and NLRP3 inflammasome activation in Coxsackievirus B3-infected cardiomyocytes

**Figure 2:**
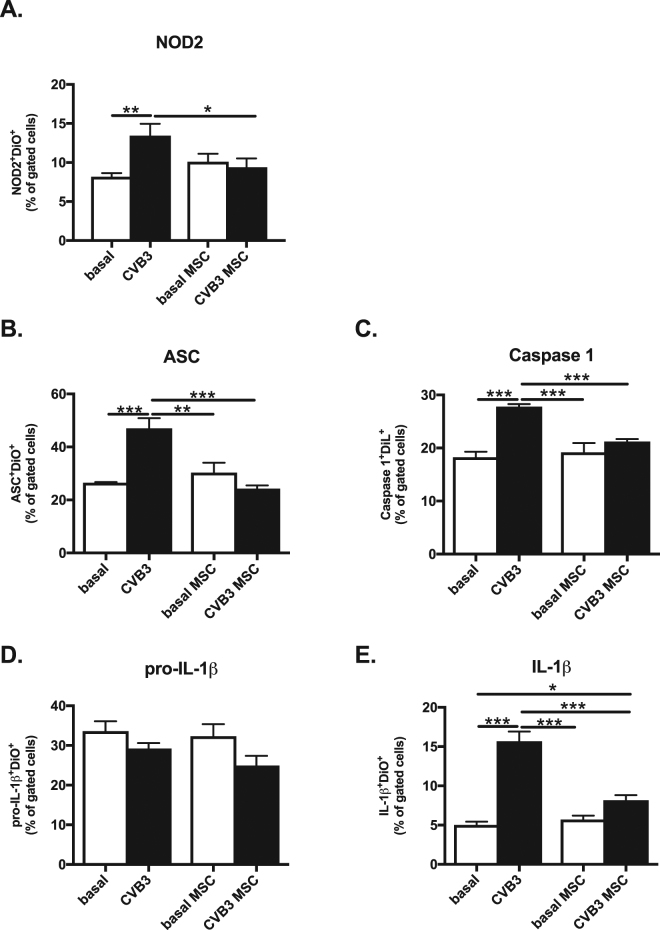
Mesenchymal stromal cells suppress NOD2 expression and NLRP3 inflammasome activation in Coxsackievirus B3-infected cardiomyocytes. Bar graphs represent the mean ± SEM of (**A**) NOD2^+^/DiO^+^, (**B**) ASC^+^/DiO^+^, (**C**) Caspase 1^+^/DiL^+^, (**D**) pro-IL-1β^+^/DiO^+^ and (**E**) IL-1β^+^/DiO^+^ cells cells depicted as the % of gated cells of basal or MSC (open bars) or CVB3-infected (closed bars) HL-1 cardiomyocytes with or without MSC, as indicated with n = 5–6/group, *p < 0.05, **p < 0.01 and ***p < 0.001.

In parallel to the *in vivo* MSC effect on NOD2 expression and NLRP3 inflammasome activation, the effect of MSC on NOD2 expression and NLRP3 inflammasome activation was evaluated *in vitro* in CVB3-infected HL-1 cardiomyocytes. MSC were added in a direct co-culture system for 4 hours to CVB3-infected Dil- or DiO-labeled HL-1 cardiomyocytes. NOD2, ASC and caspase 1 were 1.6-fold (p < 0.01), 1.7-fold (p < 0.001) and 1.6-fold (p < 0.0001) increased in CVB3-infected HL-1 cells, respectively (Fig. 2A–C). Importantly, MSC supplementation decreased NOD2- and ASC-positive CVB3-infected HL-1 cells by 1.4-fold (p < 0.05) and 1.9-fold (p < 0.0005), respectively, and diminished the downstream caspase 1 expression by 1.3-fold (p < 0.001) compared to CVB3-infected HL-1 cells. Consistent with the reduction in caspase-1 expression in HL-1 cells co-cultured with MSC, the conversion of pro-IL-1β to IL-1β declined by 1.9-fold (p < 0.0001) in CVB3-infected HL-1 cells treated with MSC (Fig. 2E), while the percentage of pro-IL-1β on its own remained unchanged in all conditions (Fig. 2D). This finding demonstrates that MSC suppress CVB3-induced NLRP3 inflammasome activation and IL-1β production in cardiomyocytes.

We have previously demonstrated that MSC exert protective effects in viral myocarditis via release of paracrine factors^18^. Oh *et al*.^20^ have recently shown that MSC efficiently inhibit NLRP3 inflammasome activation in macrophages via secretion of the anti-oxidative protein stanniocalcin (STC-1). Based on this finding and the relevance of oxidative stress in NLRP3 activation^21^, we next investigated the potential contribution of STC-1 in the observed MSC inhibitory effect on CVB3-induced NLRP3 inflammasome activation in HL-1 cells. MSC transfection with STC-1 siRNA decreased STC-1 expression in MSC by 33% (Supplemental Fig. 1A), which was translated in a modest abrogation of the inhibitory effects of MSC on NLRP3 inflammasome activity in HL-1. This follows from the observation that the % of Dil/ASC/IL-1β-positive HL-1 cells was 1.2-fold (p < 0.05) higher in HL-1 cells co-cultured with STC-1 siRNA-transfected MSC compared to HL-1 cells co-cultured with scrambled siRNA-transfected MSC (Supplemental Fig. 1B).

To study whether MSC have an effect on inflammasome activation triggered in a classical manner, we primed HL-1 cells with lipopolysaccharide (LPS) followed by adenosine-triphosphate (ATP) supplementation, which led to an increase in inflammasome activation in HL-1 cells, as evident by a 1.2-fold (p < 0.001) increase in IL-1β- and ASC-double positive HL-1 cells (Supplemental Fig. 2). Supplementation of MSC at the time of LPS priming or at the time of ATP treatment both prominently suppressed the inflammasome formation as evident by a 1.5-fold (p < 0.0001) and 2.6-fold (p < 0.0001) decrease in IL-1β- and ASC-double positive HL-1 cells, respectively (Supplemental Fig. 2).

### Mesenchymal stromal cells suppress Coxsackievirus B3-induced NOD2 expression in macrophages, natural killer cells, and dendritic cells

**Figure 3:**
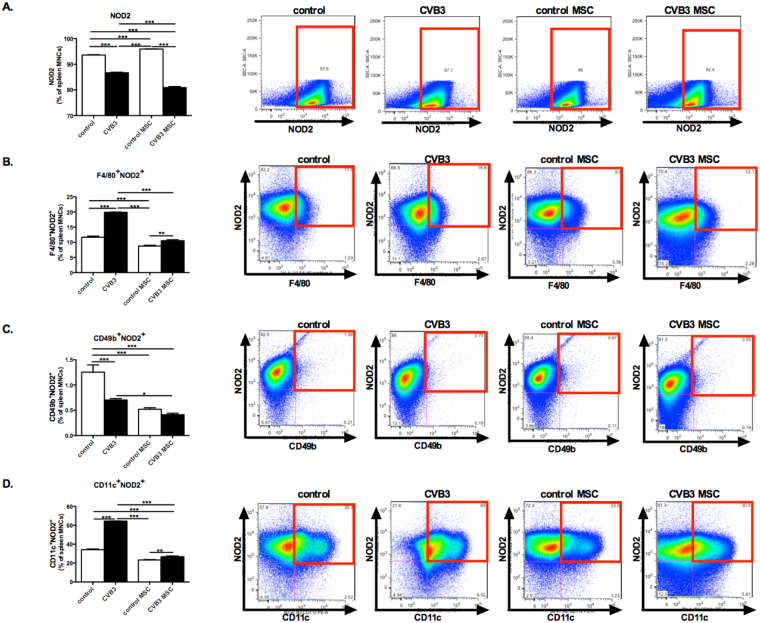
Mesenchymal stromal cells inhibit Coxsackievirus B3-induced NOD2 expression in macrophages, natural killer cells, and dendritic cells. (**A**) Bar graphs represent the mean ± SEM of NOD2-positive splenocytes in control mice (open bar) and CVB3-infected mice (closed bar) injected with PBS or MSC, expressed as the percentage of total MNCs, with n = 8–9/group. The right panel shows dot plots of NOD2 positive splenocytes of control, CVB3, control MSC, and CVB3 MSC mice, as indicated. (**B**) Bar graphs represent the mean ± SEM of F4/80^+^/NOD2^+^ in control mice (open bar) and CVB3-infected mice (closed bar) injected with PBS or MSC, expressed as the percentage of total MNCs, with n = 8–9/group and **p < 0.01. The right panel shows dot plots of F4/80^+^/NOD2^+^- positive splenocytes of control, CVB3, control MSC, and CVB3 MSC mice, as indicated. (**C**) Bar graphs represent the mean ± SEM of CD49b^+^/NOD2^+^ in control mice (open bar) and CVB3-infected mice (closed bar) injected with PBS or MSC, expressed as the percentage of total MNCs, with n = 8–9/group and **p < 0.01. The right panel shows dot plots of CD49b^+^/NOD2^+^-positive splenocytes of control, CVB3, control MSC, and CVB3 MSC mice, as indicated. (**D**) Bar graphs represent the mean ± SEM of CD11c^+^/NOD2^+^ in control mice (open bar) and CVB3-infected mice (closed bar) injected with PBS or MSC, expressed as the percentage of total MNCs, with n = 8–9/group and **p < 0.01. The right panel shows dot plots of CD11c^+^/NOD2^+^-positive splenocytes of control, CVB3, control MSC, and CVB3 MSC mice, as indicated.

Since NLRP3 is a downstream target of NOD2^4^ and a major pathogenic mediator of cardiac inflammation and dysfunction in CVB3-induced myocarditis^13^, we next investigated the effect of MSC treatment on NOD2 expression in splenic macrophages (F4/80), natural killer (NK) cells (CD49b) and DCs (CD11c) upon CVB3 infection. Splenocytes were isolated from control, control MSC, CVB3, and CVB3 MSC mice and NOD2 expression in these different immune cells populations was subsequently evaluated by flow cytometry. The total splenocytes fraction showed a 1.1-fold (p < 0.0001) increase in NOD2 expression upon CVB3 infection (Fig. 3A). In parallel, NOD2 expression was upregulated in macrophages (F4/80) and DCs (CD11c) upon CVB3 infection as indicated by a 1.2-fold (p < 0.0001) and 1.1-fold (p < 0.005) higher percentage of NOD2-positive macrophages (F4/80), and DCs (CD11c) in comparison to control mice, respectively. Importantly, MSC treatment of CVB3 mice decreased the percentage of NOD2-expressing macrophages (F4/80), NK cells (CD49b) and DCs (CD11c) by 1.2-fold (p < 0.0001), 1.1-fold (p < 0.0001) and 1.2-fold (p < 0.0001), respectively (Fig. 3B–D).

### Mesenchymal stromal cells inhibit Coxsackievirus B3-induced ASC activation in macrophages, natural killer cells, and dendritic cells

**Figure 4:**
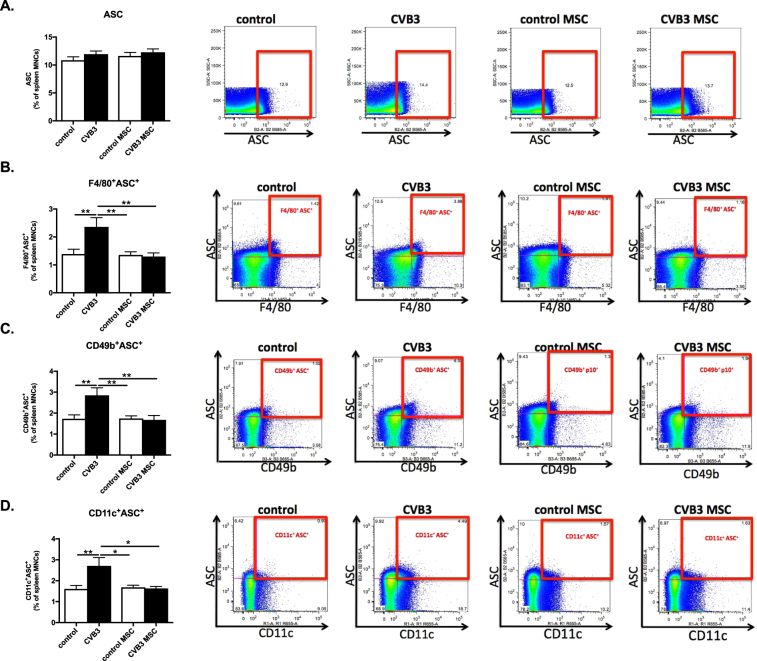
Mesenchymal stromal cells inhibit Coxsackievirus B3-induced ASC activation in macrophages, natural killer cells, and dendritic cells. (**A**) Bar graphs represent the mean ± SEM of ASC-positive splenocytes in control mice (open bar) and CVB3-infected mice (closed bar) injected with PBS or MSC, expressed as the percentage of total MNCs, with n = 8–9/group. The right panel shows dot plots of ASC positive splenocytes of control, CVB3, control MSC, and CVB3 MSC mice, as indicated. (**B**) Bar graphs represent the mean ± SEM of F4/80^+^/ASC^+^ in control mice (open bar) and CVB3-infected mice (closed bar) injected with PBS or MSC, expressed as the percentage of total MNCs, with n = 8–9/group and **p < 0.01. The right panel shows dot plots of F4/80^+^/ASC^+^- positive splenocytes of control, CVB3, control MSC, and CVB3 MSC mice, as indicated. (**C**) Bar graphs represent the mean ± SEM of CD49b^+^/ASC^+^ in control mice (open bar) and CVB3-infected mice (closed bar) injected with PBS or MSC, expressed as the percentage of total MNCs, with n = 8–9/group and **p < 0.01. The right panel shows dot plots of CD49b^+^/ASC^+^-positive splenocytes of control, CVB3, control MSC, and CVB3 MSC mice, as indicated. (**D**) Bar graphs represent the mean ± SEM of CD11c^+^/ASC^+^ in control mice (open bar) and CVB3-infected mice (closed bar) injected with PBS or MSC, expressed as the percentage of total MNCs, with n = 8–9/group and **p < 0.01. The right panel shows dot plots of CD11c^+^/ASC^+^-positive splenocytes of control, CVB3, control MSC, and CVB3 MSC mice, as indicated.

Considering the importance of ASC, which interacts with pro-caspase-1 via a CARD domain resulting in activation and maturation of caspase-1 and production of IL-1β, spleen MNCs were prepared from control, control MSC, CVB3, and CVB3 MSC mice and stained for ASC by flow cytometry. Regulation of the ASC activation was not detected when total splenocytes fraction were evaluated (Fig. 4A). However, specific immune cell subsets showed increased intracellular expression of ASC. ASC activation was induced in macrophages (F4/80), NK cells (CD49b) and DCs (CD11c) upon CVB3 infection as indicated by a 1.7-fold (p < 0.01) higher percentage of ASC in macrophages (F4/80), NK cells (CD49b) and DCs (CD11c) in comparison to control mice. In contrast, CVB3 MSC mice exhibited 1.8-fold (p < 0.01), 1.7-fold (p < 0.01) and 1.7-fold (p < 0.05) lower ASC positive macrophages, NK cells and DCs (Fig. 4B–D).

### Mesenchymal stromal cells decrease caspase-1 p10 expression in splenic macrophages, natural killer, and dendritic cells of Coxsackievirus B3-infected mice

**Figure 5:**
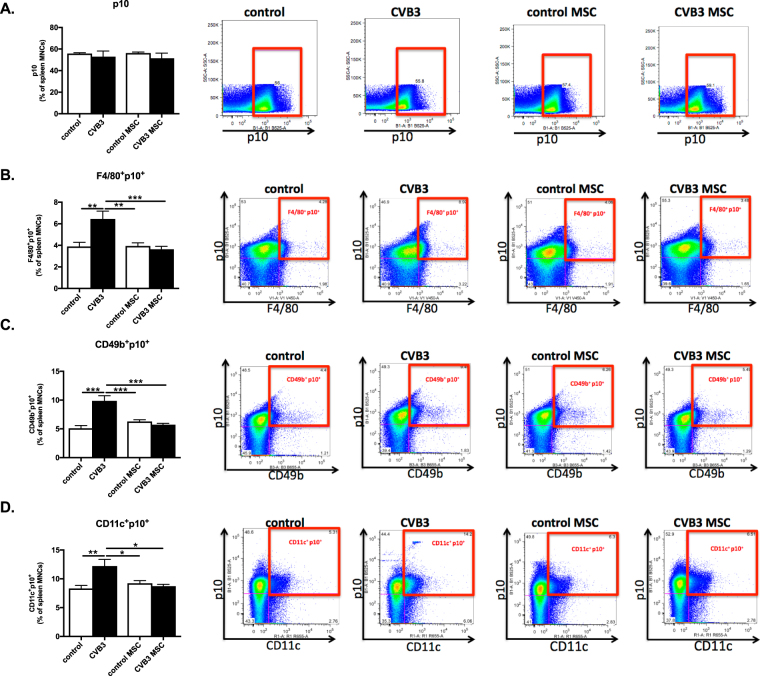
Mesenchymal stromal cells abrogate Coxsackievirus B3-induced Caspase 1 p10 induction in macrophages, natural killer cells, and dendritic cells. (**A**) Bar graphs represent the mean ± SEM of p10 positive splenocytes in control mice (open bar) and CVB3-infected mice (closed bar) injected with PBS or MSC, expressed as the percentage of total MNCs, with n = 8–9/group. The right panel shows dot plots of p10 positive splenocytes of control, CVB3, control MSC, and CVB3 MSC mice, as indicated. (**B**) Bar graphs represent the mean ± SEM of F4/80^+^/p10^+^ in control mice (open bar) and CVB3-infected mice (closed bar) injected with PBS or MSC, expressed as the percentage of total MNCs, with n = 8–9/group, **p < 0.01 and ***p < 0.001. The right panel shows dot plots of F4/80^+^/p10^+^ positive splenocytes of control, CVB3, control MSC, and CVB3 MSC mice, as indicated. (**C**) Bar graphs represent the mean ± SEM of CD49b^+^/p10^+^ in control mice (open bar) and CVB3-infected mice (closed bar) injected with PBS or MSC, expressed as the percentage of total MNCs, with n = 8–9/group and ***p < 0.001. The right panel shows dot plots of CD49b^+^/p10^+^ positive splenocytes of control, CVB3, control MSC, and CVB3 MSC mice, as indicated. (**D**) Bar graphs represent the mean ± SEM of CD11c^+^/p10^+^ in control mice (open bar) and CVB3-infected mice (closed bar) injected with PBS or MSC, expressed as the percentage of total MNCs, with n = 8–9/group, *p < 0.05 and **p < 0.01. The right panel shows dot plots of CD11c^+^/p10^+^-positive splenocytes of control, CVB3, control MSC, and CVB3 MSC mice, as indicated.

Spleen cells derived from control, control MSC, CVB3 and CVB3 MSC mice were evaluated for caspase-1 expression by measuring the intracellular levels of cleaved caspase-1 p10 subunit by flow cytometry, as a measure of caspase-1 activation. The quantification and the FACS dot plots shown in Fig. 5A illustrate no difference between the groups in caspase-1 p10 expression in the total population of spleen cells. However, CVB3-infection increased the intracellular levels of the cleaved caspase-1 p10 subunit in macrophages (F4/80), NK cells (CD49b) and DCs (CD11c) by 1.7-fold (p < 0.01), 1.9-fold (p < 0.0001) and 1.4-fold (p < 0.01), respectively, in comparison with control mice. In parallel with the effect of MSC on the initiation of NLRP3 inflammasome activation, MSC application decreased downstream p10 expression in splenic macrophages (F4/80), NK cells (CD49b), and DCs (CD11c) by 1.8-fold, 1.7-fold and 1.4-fold, respectively (Fig. 5B–D).

### Mesenchymal stromal cells abrogate secretion of IL-1β in splenic macrophages, natural killer, and dendritic cells of Coxsackievirus B3-infected mice

**Figure 6:**
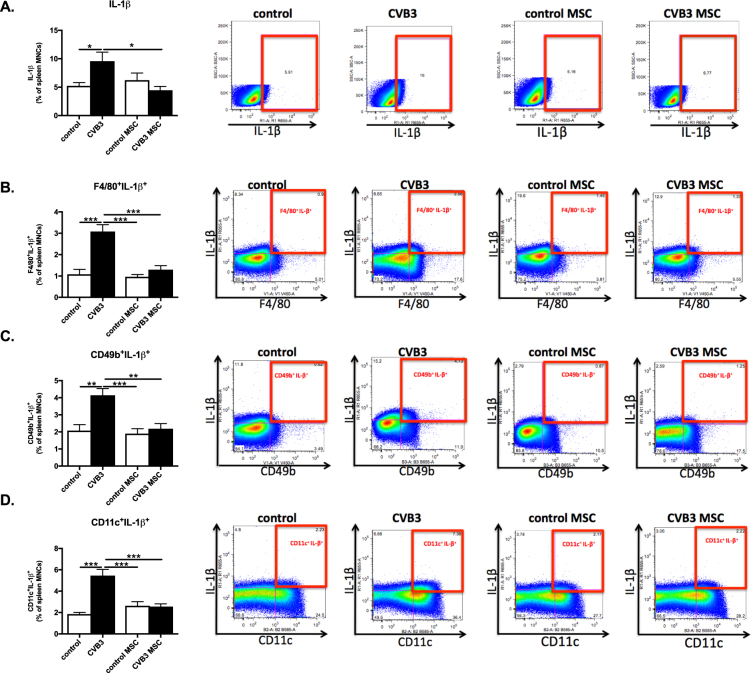
Mesenchymal stromal cells suppress Coxsackievirus B3-induced IL-1β secretion by macrophages, natural killer cells, and dendritic cells. (**A**) Bar graphs represent the mean ± SEM of IL-1β-positive splenocytes in control mice (open bar) and CVB3-infected mice (closed bar) injected with PBS or MSC, expressed as the percentage of total MNCs, with n = 8–9/group and *p < 0.05. The right panel shows dot plots of IL-1β-positive splenocytes of control, CVB3, control MSC, and CVB3 MSC mice, as indicated. (**B**) Bar graphs represent the mean ± SEM of F4/80^+^/IL-1β^+^ in control mice (open bar) and CVB3-infected mice (closed bar) injected with PBS or MSC, expressed as the percentage of total MNCs, with n = 8–9/group and ***p < 0.001. The right panel shows dot plots of F4/80^+^/IL-1β^+^ positive splenocytes of control, CVB3, control MSC, and CVB3 MSC mice, as indicated. (**C**) Bar graphs represent the mean ± SEM of CD49b^+^/IL-1β^+^ in control mice (open bar) and CVB3-infected mice (closed bar) injected with PBS or MSC, expressed as the percentage of total MNCs, with n = 8–9/group, **p < 0.01 and ***p < 0.001. The right panel shows dot plots of CD49b^+^/IL-1β^+^ positive splenocytes of control, CVB3, control MSC, and CVB3 MSC mice, as indicated. (**D**) Bar graphs represent the mean ± SEM of CD11c^+^/IL-1β^+^ in control mice (open bar) and CVB3-infected mice (closed bar) injected with PBS or MSC, expressed as the percentage of total MNCs, with n = 8–9/group and ***p < 0.001. The right panel shows dot plots of CD11c^+^/IL-1β^+^-positive splenocytes of control, CVB3, control MSC, and CVB3 MSC mice, as indicated.

IL-1β is a potent pro-inflammatory cytokine, which induces the synthesis and expression of several hundreds of secondary inflammatory mediators and it is a key mediator of the systemic inflammatory response in heart failure, having a direct cardio-depressive effect^10,11^. Spleen MNCs from CVB3-infected mice had 1.8-fold (p < 0.05) higher IL-1β expression compared to spleen MNC from controls (Fig. 6A). In parallel, IL-1β in splenic macrophages (F4/80), NK cells (CD49b) and DCs (CD11c) was 2.8-fold (p < 0.0001), 2.0-fold (p < 0.01), and 2.9-fold ( < 0.0001) higher in comparison to control mice (Fig. 6B–D). MSC application in CVB3 infected mice diminished the IL-1β releasing spleen MNCs by 2.1-fold (p < 0.05) as well as IL-1β in splenic macrophages (F4/80), NK cells (CD49b) and DCs (CD11c) by 2.4-fold (p < 0.0001), 1.9-fold (p < 0.01), and 2.1-fold (p < 0.001) (Fig. 6A–D), respectively.

To investigate the effect of MSC on classically triggered NLRP3 inflammasome activation and IL-1β secretion by splenic macrophages (F4/80), NK cells (CD49b) and DCs (CD11c), splenocytes from control mice were left untreated or primed with LPS and stimulated with ATP in the presence or absence of MSC. After 24 h of co-culture, splenocytes were collected and flow cytometry analysis was performed. LPS and ATP stimulation increased IL-1β-positive macrophages (F4/80), NK cells (CD49b) and DCs (CD11c) by 1.3-fold (p < 0.0001), 1.4-fold (p < 0.0001), and 1.2-fold (p < 0.0001), respectively. MSC induced a general suppressive effect on the LPS- and ATP-triggered IL-1β secretion of macrophages (F4/80), NK cells (CD49b) and DCs (CD11c) as evident by a 1.7-fold (p < 0.0001), 1.9-fold (p < 0.0001), and 1.5-fold (p < 0.0001) reduction in IL-1β secretion, respectively (Supplemental Fig. 3).

### Mesenchymal stromal cells application in Coxsackievirus B3-infected mice normalize the gene expression of regulators of cardiac contractility and fibrosis

**Figure 7:**
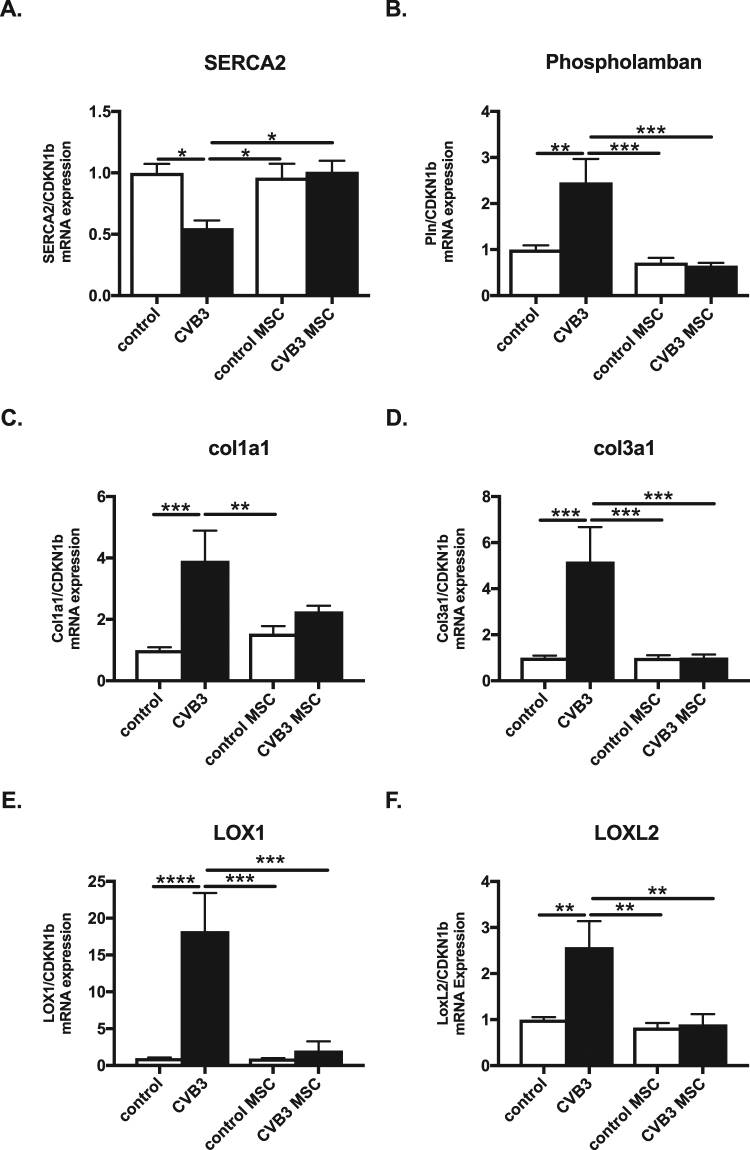
Mesenchymal stromal cells application in Coxsackievirus B3-infected mice improves the expression of genes important for cardiac contractility and fibrosis. Bar graphs represent the mean ± SEM of (**A**) SERCA2, (**B**) phospholamban, (**C**) Col1a1, (**D**) Col3a1, (**E**) LOX1 and (**F**) LOXL2 mRNA expression in the LV of control, CVB3, control MSC, and CVB3 MSC mice, as indicated, with n = 5–6/group and *p < 0.05, **p < 0.01, ***p < 0.001, and ****p < 0.0001.

Elevated IL-1β and IL-18 expression is associated with impairment of cardiac function, as shown by altered sarco/endoplasmic reticulum Ca^2+^-ATPase (SERCA2) and phospholamban expression^8^ and upregulated pro-fibrotic LOX^22^. Taking this link into account, we evaluated the LV expression of SERCA2, phospholamban, LOX1 and LOXL2, critical regulators of cardiac contractility and fibrosis, respectively, as well as of col1a1 and col3a1. CVB3 infection induced a 1.7-fold (p < 0.05) downregulation in SERCA and a 2.5-fold (p < 0.01) upregulation of phospholamban LV mRNA expression (Fig. 7A and B). MSC application in CVB3-infected mice positively regulates the gene expression of critical genes responsible for the cardiac contractility, as evident by a 1.6 fold (p < 0.05) upregulation in LV SERCA expression and a 3.8-fold (p < 0.001) downregulation in LV phospholamban expression in CVB3-infected mice treated with MSC versus CVB3 mice (Fig. 6A and B), respectively. In parallel, CVB3-infected mice exhibited 3.9-fold (p < 0.001), 5.2-fold (p < 0.001), 18-fold (p < 0.0001), and 2.6-fold (p < 0.01) higher LV col1a1, col3a1, LOX1 and LOXL2 mRNA expression, respectively, while MSC injection in CVB3 mice led to a 1.7-fold (p = 0.074), 5.1-fold (p < 0.001), 9.0-fold (p < 0.0005), and 2.9-fold (p < 0.005) lower LV col1a1, col3a1, LOX1, and LOXL2 mRNA expression, respectively (Fig. 7C–F).

The associations between cardiac contractility/cardiac fibrosis parameters and NLRP3 infammasome activation is shown by linear regressions (Supplemental Fig. 4).

## Discussion

MSC application suppresses NOD2 and NLRP3 inflammasome gene expression and prevents the NLRP3 inflammasome assembly by downregulating the ASC protein expression in the heart of the CVB3-infected mice. Moreover, the present study extends the knowledge about the systemic immunomodulatory effects of MSC in myocarditis, showing that MSC abrogate the expression of NOD2, NLRP3 inflammasome activation and subsequent IL-1β production in macrophages (F4/80), NK cells (CD49b) and DCs (CD11c) of CVB3-infected mice. The suppressive effect of MSC on NOD2 expression and NLRP3 inflammasome activation is translated into normalized expression of important genes of cardiac contractility and fibrosis, which could potently abrogate the development of adverse cardiac dysfunction and remodelling following viral-induced myocardial injury. We suggest that the inhibitory effects of MSC on NLRP3 inflammasome activation is mediated at least partly via suppression of NOD2 expression and via secretion of anti-oxidative STC-1.

NLRP3 inflammasome activation is detected in heart biopsy samples of acute myocarditis patients and postmortem cases of myocarditis of unknown etiology^23^ and plays a leading role in the pathogenesis of myocarditis^12^. NLRP3 activation can be due to NLRP3 priming, i.e. induction of NLRP3 mRNA expression involving NF-kB or upstream NOD2, or due to direct activation of the NLRP3 inflammasome via mediators like reactive oxygen species (ROS) and ATP^4^. NOD2 is a cytoplasmatic PRR, which recognizes ssRNA, including CVB3^5^ and activates NLRP3 inflammasome and IL-1β processing and secretion^4^. Our recent findings reveal that NOD2 is a major mediator in the pathogenesis of CVB3-induced myocarditis^13^. We showed that NOD2 expression was upregulated in endomyocardial biopsies of CVB3-positive patients. Furthermore, cardiac NOD2 and NLRP3 expression dropped in CVB3-positive patients who eliminated the virus and improved cardiac function over time^13^. The relevance of NOD2 expression in CVB3 myocarditis further followed from NOD2^−/−^ CVB3 mice which were rescued from the detrimental effects of CVB3. They exhibited a minor inflammatory response, lower cardiac fibrosis and decreased NLRP3 activation. It should be noted however that the NOD2 expression was not increased in patients with a persistence of the cardiotropic viruses parvovirus B19 or human herpesvirus 6 (HHV-6) type B, which are among the most frequently found cardiotropic viruses in endomyocardial biopsies^24–26^, whereas its regulation in primarily autoimmune myocarditis still needs to be investigated. Our present findings which indicate a potent inhibitory effect of MSC on cardiac CVB3-induced NOD2 gene expression might therefore only be limited to the pathology of CVB3-induced myocarditis, and not generalized for all myocarditis etiologies. MSC further reduced the gene expression of all NLRP3 inflammasome components and products, which relevance has been shown in different cardiovascular disorders besides myocarditis^11,27^. Only ASC protein level was markedly increased in the heart of CVB3-infected mice and downregulated by MSC, while at this time point, cardiac caspase 1 activity and IL-1ß protein expression remained unchanged upon CVB3 infection and were not affected by MSC application. Interestingly, *see supra*, caspase 1 and IL-1ß protein expression was already increased in splenic macrophages, NK cells and DCs of those CVB3-infected versus control mice. The observed discrepancy in caspase 1 and IL-1β protein expression in the heart and the spleen at the same time point probably reflects a difference in the basal expression of NLRP3 inflammasome and in the mechanism of inflammasome activation. Tissue resident cells may not need to respond to injurious/infectious agents as promptly as cells of the innate immune response. Indeed, the mechanism of inflammasome regulation has been shown to be tissue-specific as NLRP3 expression in the heart of wildtype mice is lower than that in the spleen^28^. Nevertheless, the upregulated LV protein expression of ASC in CVB3-infected mice indicates already initiation of NLRP3 inflammasome formation^4^ in the heart of myocarditis mice, which could be decreased via MSC application. The suppressive effect of MSC on NOD2, NLRP3 inflammasome and IL-1β expression was confirmed *in vitro* in CVB3-infected cardiomyocytes. MSC supplementation abolished not only the CVB3-induced ASC protein expression and NLRP3 assembly, but also the downstream caspase 1 expression and conversion of pro-IL-1β to active IL-1β in CVB3-infected HL-1 cells.

ROS are a central and common upstream cellular signal triggering NLRP3 inflammasome activation^21^. The intracellular oxidation status has been implicated in the pathogenesis of CVB3 infections^29^. Moreover, Wang *et al*.^12^ showed that the production of ROS is crucial for inflammasome activation in response to CVB3 infection. Importantly, we have previously demonstrated that MSC reduce the CVB3-induced ROS production in cardiomyocytes^18^, whereas Oh *et al*.^20^ recently illustrated that MSC decrease mitochondrial ROS and inhibit LPS- and ATP-induced NLRP3 inflammasome activation in macrophages primarily by secreting STC-1. Based on these findings, we wanted to understand if MSC negatively regulate the NLRP3 inflammasome activation in CVB3-infected HL-1 cells in an STC-1-dependent manner. Therefore, we knocked down STC-1 in MSC and subsequently co-cultured them with CVB3-infected HL-1 cardiomyocytes. The partial knockdown of STC-1 in MSC modestly abrogated the inhibitory effect on NLRP3 activation in cardiomyocytes (Supplemental Fig. 1) suggesting that MSC mediate their effect at least partly in an STC-1-dependent manner. Furthermore, we demonstrated that MSC reduce LPS- and ATP-triggered NLRP3 inflammasome activity in HL-1 cardiomyocytes, indicating that the inhibitory effect on the NLRP3 inflammasome is not solely restricted to CVB3 as a trigger, a finding which could also be confirmed in splenic macrophages, NK cells, and DCs. Taken together the discussed findings suggest that MSC suppress NLRP3 inflammasome activation presumably via reduction of NOD2 expression, ROS production and release of paracrine factors, such as the anti-oxidative factor STC-1.

Aside of the direct cardiac injury in CVB3-induced myocarditis^30^, inflammation is the leading component and the dominant mechanism in the pathogenesis of viral myocarditis^1^. MSC have been shown to inhibit NLRP3 inflammasome activation *in vitro* in macrophages^20^. In the present study, we could demonstrate that MSC mediate an effective systemic immunoregulation in viral myocarditis via reduction of NOD2 expression and abrogation of NLRP3 inflammasome activation in macrophages (F4/80), NK cells (CD49b) and DCs (CD11c). NOD2 activation in inflammatory cells may induces NF-κB and MAPK signalling pathways triggering a cell signaling cascade enhancing the secretion of pro-inflammatory immune factors - TNF-α, IL-6, CCL2, IL-8 -^31^, which affect signaling molecules in cardiac residential cells ultimately resulting in cardiac injury. NOD2 induces the production of CC-chemokine ligand 2 (CCL2) and helps to drive the recruitment and priming of innate immune cells, including neutrophils and inflammatory Ly6C^hi^ monocytes^32^. Importantly, we have recently demonstrated that MSC attenuate myocardial inflammation via suppression of the cardiac infiltration of Ly6C^hi^ pro‐inflammatory monocytes in CVB3-induced myocarditis^33^. Furthermore, NOD2 supports the recruitment of inflammatory macrophages^34^, known to mediate deteriorating immune overreaction as depletion of macrophages significantly improve both acute and chronic viral myocarditis, further confirming the critical pathological role of macrophages in CVB3-induced myocarditis^35^. Macrophages are the most abundant cells in the inflamed myocardium of CVB3-infected mice and induce the expression of IL-1β, promoting cardiac inflammation, cardiac dilatation, fibrosis and heart failure^36^. Therefore, we assume that the suppressive effect of MSC on NLRP3 inflammasome activation and IL-1β secretion by macrophages could be beneficial in viral myocarditis preventing disease progression to cardiac dilatation and heart failure. DCs infiltrate the myocardium and release a cocktail of proinflammatory cytokines and induce CD4^+^ T-cell-mediated autoimmune induction in experimental CVB3 myocarditis^37^. MSC inhibit the NLRP3 inflammasome activation and IL-1β release by DCs, which might prevent autoimmunity induction. Given the significant involvement of autoimmune mechanisms in both experimental^38,39^ and human^40^ myocarditis and the finding that excessive inflammasome activation can cause autoimmune disorders^41^, these inhibitory effects of MSC on inflammasome activation could have major implications on autoimmune reactivity in CVB3 myocarditis which could subsequently lead to an improvement in chronic myocarditis pathology.

The NK cells not only control the virus replication^42^, but also release perforin in CVB3-induced myocarditis^43^. MSC inhibit the NLRP3 inflammasome activation and IL-1β released by NK cells, limiting the long-term NK cells activation and release of cytotoxic factors which could damage myocytes in absence of significant virus titers in the heart tissue.

IL-1β levels strongly correlate with the severity of CVB3-induced myocarditis^12^. MSC treatment in CVB3 mice downregulated LV mRNA expression of IL-1β and IL-18, which both induce cardiodepressive effects in heart failure^10,11^ and alter phospholamban expression, a key regulator of cardiac contractility^8^. Blockade of IL-1β or neutralization of IL-18 is an effective cardioprotective approach^10,44^. Therefore, the inhibitory effect of MSC on LV IL-1β and IL-18 gene expression and IL-1β-expressing splenic macrophages, NK cell, and DCs could have beneficial cardiac therapeutic implications.

In agreement with the previously demonstrated anti-fibrotic and cardioprotective effects of MSC, associated with improved LV function in CVB3-infected myocarditis mice as demonstrated via conductance hemodynamic measurments^18,19^, we now show in the same experimental setting that the MSC inhibitory effect on NOD2 and NLRP3 inflammasome activation is associated with an improved expression of markers involved in Ca^2+^ regulation (SERCA, phospholamban) and cardiac fibrosis (col3a1, LOX1, LOXL2). Decreased expression levels of SERCA and a lower ratio of SERCA to its endogenous modulator phospholamban in the heart results in decreased contractility^45^, whereas LOX enzymes are key players in extracellular matrix deposition, maturation and cross-linking of collagen fibrils, which leads to myocardial stiffness, left ventricular dysfunction, and heart failure^46,47^. Moreover, we found a strong correlation between LV NLRP3 and phospholamban, col3a1, LOX1, and LOXL2 mRNA expression (Supplemental Fig. 4). These observations further support and clarify that i.v. MSC application improves LV function (contractility, stiffness) in CVB3 myocarditis mice via reducing cardiac fibrosis (collagen and crosslinking) and via modulating the expression of proteins involved in Ca^2+^ regulation.

**Figure 8:**
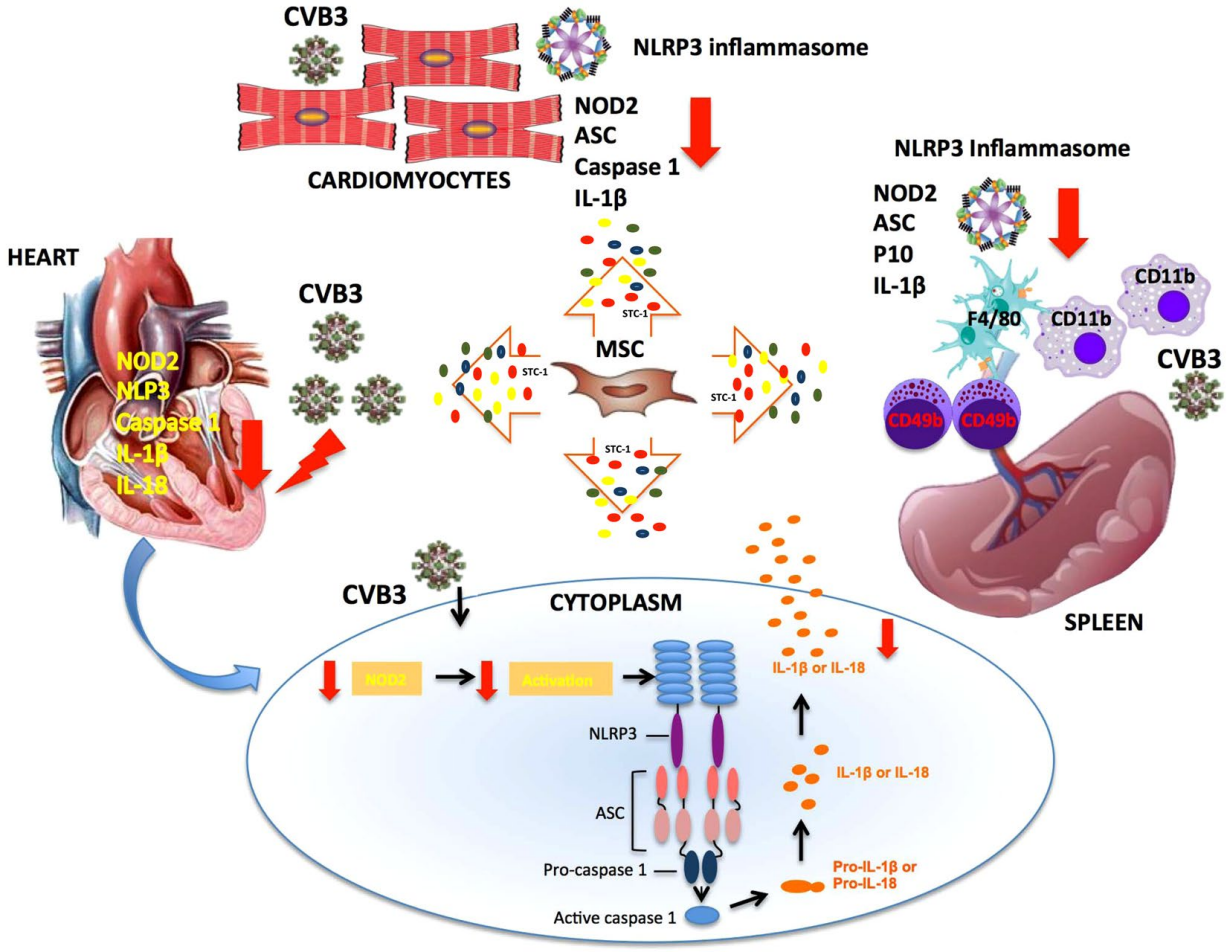
Hypothetical scheme of the effect of MSC application on NLRP3 inflammasome activation in the heart and innate immune cells of Coxsackievirus B3-infected mice. MSC application abrogates the expression of NOD2 and NLRP3 inflammasome components NLRP3, ASC, caspase 1 and IL-1β and IL-18 in the heart of CVB3-infected mice. The inhibitory effect of MSC on CVB3-induced inflammasome activation in cardiomyocytes was confirmed *in vitro* as indicated by a reduction in NOD2-, ASC-, and caspase 1-positve CVB3-infected cardiomyocytes and subsequent decrease in IL-1β secretion in the presence of MSC. MSC application mediated a systemic immunoregulation by suppressing NOD2 expression, inhibiting NLRP3 inflammasome activation and IL-1β secretion by macrophages, natural killer cells, and dendritic cells. MSC exert inhibitory effects on NLRP3 inflmmasome activation presumably partly via suppressing NOD2 expression and partly via secretion of the paracrine factor STC-1. MSC application is an effective strategy abrogating NLRP3 inflammasome activation and preventing the adverse consequences of excessive myocardial and systemic inflammation. All MSC-mediated effects are marked with a red arrow. STC-1 is marked as a red bullet.

In summary, the present study demonstrates that MSC are potent regulators of the NLRP3 inflammasome in the heart and in innate immune cells. MSC decrease NLRP3 activity, presumably from the combined reduction in NOD2 expression and the decrease in NLRP3 inflammasome activity involving the paracrine factor STC-1 (Fig. 8). Therefore, MSC application could be a promising strategy to hamper the NLRP3 inflammasome activation limiting the adverse consequences of excessive myocardial and systemic inflammation associated with abnormal expression of genes regulators of cardiac contractility and fibrosis.

## Methods

### Murine Coxsackievirus B3-induced myocarditis and cell application

Eight-week-old male C57BL/6 mice were infected by intraperitoneal injection of 1 × 10^5^ plaque forming units (PFU) of CVB3 virus (Nancy strain). Control mice received PBS instead of CVB3. One day after CVB3 virus infection, 1 × 10^6^ MSC were i.v. administrated via the tail vein into C57BL/6 mice. All mice were sacrificed on day seven post-CVB3 infection. The LV was harvested and snap-frozen for molecular biology. For flow cytometry analysis, heart, blood, and spleen mononuclear cells (MNCs) were isolated. The investigation was approved by the local ethical committee (Landesamt für Gesundheit und Soziales, Nr: G0094/11) and was performed in accordance with the European principles of laboratory animal care (Directive 2010/63/EU) and German animal protection law.

### Mesenchymal Stromal Cell Isolation

Human adult MSC were isolated from iliac crest bone marrow aspirates of 8 healthy donors after their written approval according to Binger *et al*.^48^ MSC were cultured and expanded for injection in Dulbecco’s Modified Eagle’s Medium (Biochrom, Berlin, Germany) supplemented with 10% fetal bovine serum (FBS), 1% penicillin/streptomycin, 1% glutamine, 2% HEPES, and 2 ng/mL of basic fibroblast growth factor (Tebu-bio, Offenbach, Germany). Cultivated MSC were triple negative for the markers CD45, CD34, and CD11b, but stained positively for the markers CD73, CD29, CD105, CD106, CD90, and CD44.

### Spleen mononuclear cell isolation and flow cytometry analysis

Splenocytes were isolated from control PBS, control MSC, CVB3 and CVB3 MSC mice according to Van Linthout *et al*.^18^. All samples were pre-incubated with FcR block Ab and flow cytometry analysis of spleen MNCs was performed using directly conjugated monoclonal mouse antibodies: anti-mouse F4/80 Pacific Blue (BM8), anti-mouse CD49b PerCP/Cy5.5 (DX5), CD11c APC (N418) or CD11c PE (N418), (Biolegend San Diego, CA, USA). Surface staining was performed according to the manufacturer’s instructions. IL-1β in spleen MNCs was measured after 12 h incubation with 50 ng/ml phorbol myristate acetate (PMA) and 500 ng/ml ionomycin in the presence of BD GolgiStop™ Protein Transport Inhibitor (BD Biosciences, Franklin Lakes, NJ, USA). For intracellular staining, the MNCs were fixed and permeabilized for 20 min at 4 °C with Cytoperm/Cytofix (BD Biosciences, Franklin Lakes, NJ, USA) and stained with anti-mouse NOD2 APC (R&D Systems Europe Ltd., Abingdon, OX14 3NB, United Kingdom), anti-mouse IL-1β APC (Monoclonal Rat IgG2B Clone #166931) (R&D Systems, Minneapolis, MN, USA), caspase-1 p10 Alexa Fluor 488 (Antibodies online, Atlanta, GA, USA) and ASC PE (Biolegend, San Diego, CA, USA) antibodies in BD Perm/Wash solution according to the manufacturer’s instructions. Sample analysis was performed on a MACSQuant Analyzer (Miltenyi Biotec, Bergisch Gladbach, Germany) and flow cytometry data were analyzed with FlowJo 8.7. software (FlowJo, LLC, RO, USA).

### Gene expression analysis

Frozen murine heart tissue was homogenized with an IKA T25D ULTRA TURRAY homogenisator (Laboratory equipment, Germany) in Trizol, followed by chloroform extraction and isopropanol precipitation. MSC were collected 48 h post siRNA transfection in Trizol for RNA preparation. Next, RNA was DNase treated with the NucleoSpin RNA II Kit (Macherey-Nagel, Düren, Germany) and subsequently reverse transcribed via High Capacity cDNA Reverse Transcription Kit from Applied Biosystems by Thermo Fisher Scientific (Carlsbad, CA, USA). To assess the mRNA expression of the murine target genes *NLRP3*, *ASC*, *caspase 1*, *IL-1β*, *IL-18*, *SERCA2*, *phospholamban*, *collagen 1* (*col1a1*), *collagen 3* (*col3a1*), *LOX*, and *LOXL2*, and human *stanniocalcin* (*STC*)*-1*, mRNA expression was analyzed via real-time PCR using gene expression assays for NLRP3 Mm00840904_m1, ASC Mm00445747_g1, caspase 1 Mm00438023_m1, IL-1β Mm00434228_m1, IL-18 Mm00434225_m1, SERCA2 Mm01201431_m1, phospholamban Mm04206542_m1, col1a1 Mm01302043_g1, col3a1 Mm00802331_m1, LOX Mm00495386_m1 and LOXL2 Mm00804740_m1, and STC-1 Hs00174970_m1 from Applied Biosystems by Thermo Fisher Scientific (Carlsbad, CA, USA). Murine mRNA expression was normalized to the housekeeping gene CDKN1b Mm00438167_g1 and relatively expressed with the control group set as 1. Human STC-1 was normalized to the housekeeping gene CDKN1b Hs00153277_m1 and relatively expressed with the control group set as 100%.

### HL-1 and mesenchymal stromal cell co-culture experiments

Murine HL-1 cells were cultured in Claycomb medium (Sigma-Aldrich, St. Louis, MO, USA) supplemented with 10% FBS, 1% penicillin/streptomycin, 100 μM norepinephrine (Sigma-Aldrich, St. Louis, MO, USA) and 2 mM glutamine (Biochrom, Berlin, Germany) for CVB3 infection and LPS and ATP stimulation experiments. For both experiments, DiO- or Dil-labeled (Vybrant® DiO or DiL Cell-labeling solution, Applied Biosystems by Thermo Fisher Scientific, Carlsbad, CA, USA) HL-1 were plated into 6-well plates at a density of 250,000 cells/6-well for ASC, caspase 1, IL-1β or pro-IL-1β flow cytometry analysis, respectively.

For CVB3 infection experiments, DiO- or Dil-labeled HL-1 cells were 24 h after plating, serum starved or infected with CVB3 under serum starvation conditions at a m.o.i. of 4 for 1 h. Then, cells were washed with PBS two times and complete Claycomb medium was added. Untreated MSC or MSC 48 h after tranfection with 30 nM scrambled or stanniocalcin (STC-1) siRNA were collected and added to the HL-1 cells for co-culture at a ratio of MSC to HL-1 of 1 to 10, which is a commonly used ratio when MSCs are co-cultured in the presence of a target cell of interest^18^. Four h after infection, cells were collected for flow cytometry purposes.

For LPS and ATP stimulation experiments, DiO-labeled HL-1 cells were washed with PBS 24 h after plating and next stimulated with 25 ng/ml of LPS from *Escherichia coli* O55:B5 (Merck) for 2 h. Then, cells were washed twice with PBS and stimulated with 5 mM ATP for 1 h (Invivo Gen, San Diego, CA, USA). MSC were added for co-culture at a ratio of 1 MSC to 10 HL-1 at the timepoint of LPS or ATP administration. One h after ATP supplementation, cells were collected for flow cytometry purposes.

### Splenocytes and mesenchymal stromal cell co-culture experiments

MSC were plated at a density of 15,000 cells/96-well in complete MSC medium. Twenty-four h later, splenocytes from control mice were added at a ratio of 10 to 1 MSC in 90 µl of RPMI1640 medium supplemented with 10% FBS and 1% penicillin/streptomycin with/out 25 ng/ml LPS (Merck). After 2 h, 0 or 10 µl of ATP was added to the LPS conditions at a final conc. of 5 mM, whereas to the other wells 10 µl of PBS was added. After 1 h, cells were fixed and permeabilized for 20 min at 4 °C with Cytoperm/Cytofix (BD Biosciences, Franklin Lakes, NJ, USA) and stained with anti-mouse F4/80 Pacific Blue (BM8), anti-mouse CD49b PerCP/Cy5.5 (DX5), CD11c APC (N418) or CD11c PE (N418) (Biolegend), anti-mouse ASC PE (Biolegend) and anti-mouse IL-1β APC (Monoclonal Rat IgG2B Clone #166931) (R&D Systems) antibodies in BD Perm/Wash solution according to the manufacturer’s instructions. Sample analysis was performed on a MACSQuant Analyzer (Miltenyi Biotec, Bergisch Gladbach, Germany) and flow cytometry data were analyzed with FlowJo 8.7. software (FlowJo, LLC, RO, USA).

### Flow cytometry analysis of NOD2, apoptosis-associated speck-like protein containing a CARD, IL-1β and pro-IL-1β

DiO-labeled HL-1 cells were infected with CVB3 and subsequently co-cultured with MSC as described above. Four hours post CVB3 infection, HL-1 cells were fixed in Fixation/Permeabilization solution (BD Biosciences, Franklin Lakes, NJ, USA) and stained with APC anti-NOD2 (R&D Systems Europe Ltd.), PE anti-ASC (TMS-1) (Biolegend San Diego, CA, USA), anti-mouse PerCP mIL-1β/IL-1F2 (R&D Systems, Minneapolis, MN, USA) or anti-mouse PE IL-1β pro-form (eBioscience, San Diego, CA, USA). Sample analysis was performed on a MACSQuant Analyzer (Miltenyi Biotec, Bergisch Gladbach, Germany) and flow cytometry data were analyzed with FlowJo 8.7. software (FlowJo, LLC, RO, USA). Data are presented as DiO^**+**^ASC^**+**^ cells (% of gated) or DiO^**+**^IL-1β^**+**^ (% of gated) or DiO^**+**^pro-IL-1β^**+**^ cells (% of gated), respectively.

### Flow cytometry analysis of caspase 1

DiL-labeled HL-1 cells were infected with CVB3 and subsequently co-cultured with MSC as described above. Four hours post CVB3 infection HL-1 cells were collected and stained for flow cytometry analysis with Caspase-1 FAM-YVAD- FMK KIT (ImmunoChemistry Technologies, Bloomington, U.S.A.) according to the manufacturer’s instruction and washed with 1× Apoptosis Wash Buffer. Sample analysis was performed on a MACSQuant Analyzer (Miltenyi Biotec, Bergisch Gladbach, Germany) and flow cytometry data were analyzed with FlowJo 8.7. software (FlowJo, LLC, RO, USA). Data are presented as DiL^**+**^Caspase 1 cells^**+**^ (% of gated).

### Immunohistology

Immunohistochemistry was carried out on 5 µm-thick cryosections using ASC (TMS-1) antibody (GeneTex, CA, USA). Analysis of stained sections was made in a blinded fashion by digital image analysis on a Leica DZ 2000 LED microscope (Leica Microsystems, Wetzlar, Germay) at a 100× magnification.

### Caspase-1 activity assay

LV caspase 1 activity was measured with a caspase-1 colorimetric assay (R&D Systems, Minneapolis, MN, USA) according to the manufacturer’s protocol. In brief, 50 µl of tissue lysate containing 100 µg of LV protein extract of C57BL/6 control and CVB3-infected mice i.v. injected with MSC or PBS was mixed with 50 μl of 2X Reaction Buffer 1 and 5 μl of a caspase-1 colorimetric substrate and incubated for 2 hours at 37 °C. The caspase 1 activity in the samples was quantified with a microplate reader using a wavelength of 405 nm. Data represent the absorbance of the samples.

### IL-1β ELISA

Mouse cardiac IL-1β levels were determined in tissue lysate of LV protein extract (diluted 1:150, total volume 100 µl) of C57BL/6 control and CVB3-infected mice i.v. injected with MSC or PBS. A mouse IL-1β ELISA kit (Cloud Clone Corp., Houston, USA) was used according to the manufacturer’s protocol. IL-1ß concentrations were normalized to total LV protein concentrations.

### siRNA experiments

MSC were transfected with siPORT™ Amine Transfection Agent (Thermo Fisher Scientific, Waltham, Massachusetts, USA) with 30 nM of STC-1 or scrambled siRNA (Thermo Fisher Scientific) following the manufacturer’s protocol. Real-time PCR was performed to confirm knockdown of STC-1 (Supplemental Fig. 1A).

### Statistical analysis

Statistical analysis was performed using Prism 6 for Mac OS X (GraphPad Software, Inc., La Jolla, USA). Ordinary one-way ANOVA was used for statistical analysis of the data with correction for multiple comparisons via the Tukey test. Data are presented as mean ± SEM. Differences were considered to be significant when the two-sided p-value was lower than 0.05.

## Electronic supplementary material

Supplementary Information

## References

[1] Caforio AL (2013). Current state of knowledge on aetiology, diagnosis, management, and therapy of myocarditis: a position statement of the European Society of Cardiology Working Group on Myocardial and Pericardial Diseases. Eur Heart J..

[2] Janeway CA, Medzhitov R (2002). Innate immune recognition. Annu Rev Immunol..

[3] Müller, I. *et al*. Pathogenic role of the damage-associated molecular patterns S100A8 and S100A9 in Coxsackievirus B3-induced myocarditis. *Circ Heart Fail.*; In Press (2017). 10.1161/CIRCHEARTFAILURE.117.004125 29158436

[4] Sutterwala FS, Haasken S, Cassel SL (2014). Mechanism of NLRP3 inflammasome activation. Ann N Y Acad Sci..

[5] Sabbah A (2009). Activation of innate immune antiviral responses by Nod2. Nat Immunol..

[6] Li X (2013). NOD2 deficiency protects against cardiac remodeling after myocardial infarction in mice. Cell Physiol Biochem..

[7] Van Tassell BW, Toldo S, Mezzaroma E, Abbate A (2013). Targeting interleukin-1 in heart disease. Circulation..

[8] McTiernan CF (1997). Interleukin-1 beta inhibits phospholamban gene expression in cultured cardiomyocytes. Circ Res..

[9] Pomerantz BJ, Reznikov LL, Harken AH, Dinarello CA (2001). Inhibition of caspase 1 reduces human myocardial ischemic dysfunction via inhibition of IL-18 and IL-1beta. Proc Natl Acad Sci USA.

[10] Abbate A (2008). Anakinra, a recombinant human interleukin-1 receptor antagonist, inhibits apoptosis in experimental acute myocardial infarction. Circulation..

[11] Kawaguchi M (2011). Inflammasome activation of cardiac fibroblasts is essential for myocardial ischemia/reperfusion injury. Circulation..

[12] Wang Y, Gao B, Xiong S (2014). Involvement of NLRP3 inflammasome in CVB3-induced viral myocarditis. Am J Physiol Heart Circ Physiol..

[13] Tschope, C. *et al*. NOD2 (Nucleotide-Binding Oligomerization Domain 2) Is a Major Pathogenic Mediator of Coxsackievirus B3-Induced Myocarditis. *Circ Heart Fail.* **10** (2017). 10.1161/CIRCHEARTFAILURE.117.003870 28912259

[14] Ridker PM (2017). Antiinflammatory Therapy with Canakinumab for Atherosclerotic Disease. N Engl J Med..

[15] Miteva K, Van Linthout S, Volk HD, Tschope C (2013). Immunomodulatory effects of mesenchymal stromal cells revisited in the context of inflammatory cardiomyopathy. Stem Cells Int..

[16] Tschope C, Miteva K, Schultheiss HP, Linthout SV (2011). Mesenchymal stromal cells: a promising cell source for the treatment of inflammatory cardiomyopathy. Curr Pharm Des..

[17] Van Linthout, S. *et al*. Placenta-Derived Adherent Stromal Cells Improve Diabetes Mellitus-Associated Left Ventricular Diastolic Performance. *Stem Cells Transl Med* (2017). 10.1002/sctm.17-0130 PMC5702519 29024485

[18] Van Linthout S (2011). Mesenchymal stem cells improve murine acute coxsackievirus B3-induced myocarditis. Eur Heart J..

[19] Savvatis K (2012). Mesenchymal stromal cells but not cardiac fibroblasts exert beneficial systemic immunomodulatory effects in experimental myocarditis. PLOS One..

[20] Oh JY (2014). Mesenchymal stem/stromal cells inhibit the NLRP3 inflammasome by decreasing mitochondrial reactive oxygen species. Stem Cells..

[21] Martinon F (2010). Signaling by ROS drives inflammasome activation. Eur J Immunol..

[22] Hofnagel O (2004). Proinflammatory cytokines regulate LOX-1 expression in vascular smooth muscle cells. Arterioscler Thromb Vasc Biol..

[23] Toldo S (2014). Formation of the inflammasome in acute myocarditis. Int J Cardiol..

[24] Kuhl U (2005). High prevalence of viral genomes and multiple viral infections in the myocardium of adults with “idiopathic” left ventricular dysfunction. Circulation..

[25] Kuhl U (2005). Viral persistence in the myocardium is associated with progressive cardiac dysfunction. Circulation..

[26] Andreoletti L (2009). Viral causes of human myocarditis. Arch Cardiovasc Dis..

[27] Mezzaroma E (2011). The inflammasome promotes adverse cardiac remodeling following acute myocardial infarction in the mouse. Proc Natl Acad Sci USA.

[28] Lech M (2010). Quantitative expression of RIG-like helicase, NOD-like receptor and inflammasome-related mRNAs in humans and mice. Int Immunol..

[29] Si X (2005). Pyrrolidine dithiocarbamate reduces coxsackievirus B3 replication through inhibition of the ubiquitin-proteasome pathway. J Virol..

[30] Badorff C (2000). Nitric oxide inhibits dystrophin proteolysis by coxsackieviral protease 2A through S-nitrosylation: A protective mechanism against enteroviral cardiomyopathy. Circulation..

[31] Fritz JH (2005). Synergistic stimulation of human monocytes and dendritic cells by Toll-like receptor 4 and NOD1- and NOD2-activating agonists. Eur J Immunol..

[32] Coulombe F (2012). Muramyl dipeptide induces NOD2-dependent Ly6C(high) monocyte recruitment to the lungs and protects against influenza virus infection. PLoS One..

[33] Miteva, K. *et al*. Mesenchymal Stromal Cells Modulate Monocytes Trafficking in Coxsackievirus B3-Induced Myocarditis. *Stem Cells Transl Med* (2017). 10.1002/sctm.16-0353 PMC5442851 28186704

[34] Davis KM, Nakamura S, Weiser JN (2011). Nod2 sensing of lysozyme-digested peptidoglycan promotes macrophage recruitment and clearance of S. pneumoniae colonization in mice. J Clin Invest..

[35] Liu L, Yue Y, Xiong S (2014). NK-derived IFN-gamma/IL-4 triggers the sexually disparate polarization of macrophages in CVB3-induced myocarditis. J Mol Cell Cardiol..

[36] Li K (2009). Differential macrophage polarization in male and female BALB/c mice infected with coxsackievirus B3 defines susceptibility to viral myocarditis. Circ Res..

[37] Eriksson U (2003). Dendritic cell-induced autoimmune heart failure requires cooperation between adaptive and innate immunity. Nat Med..

[38] Neu N (1987). Autoantibodies specific for the cardiac myosin isoform are found in mice susceptible to Coxsackievirus B3-induced myocarditis. J Immunol..

[39] Neu N (1987). Coxsackievirus induced myocarditis in mice: cardiac myosin autoantibodies do not cross-react with the virus. Clin Exp Immunol..

[40] Neumann DA (1990). Circulating heart-reactive antibodies in patients with myocarditis or cardiomyopathy. J Am Coll Cardiol..

[41] Shaw PJ, McDermott MF, Kanneganti TD (2011). Inflammasomes and autoimmunity. Trends Mol Med..

[42] Ong S (2015). Natural killer cells limit cardiac inflammation and fibrosis by halting eosinophil infiltration. Am J Pathol..

[43] Escher F (2014). Presence of perforin in endomyocardial biopsies of patients with inflammatory cardiomyopathy predicts poor outcome. Eur J Heart Fail..

[44] Abbate A (2010). Interleukin-1 blockade with anakinra to prevent adverse cardiac remodeling after acute myocardial infarction (Virginia Commonwealth University Anakinra Remodeling Trial [VCU-ART] Pilot study). Am J Cardiol..

[45] Kiss E, Jakab G, Kranias EG, Edes I (1994). Thyroid hormone-induced alterations in phospholamban protein expression. Regulatory effects on sarcoplasmic reticulum Ca2+ transport and myocardial relaxation. Circ Res..

[46] Kasner M (2011). Diastolic tissue Doppler indexes correlate with the degree of collagen expression and cross-linking in heart failure and normal ejection fraction. J Am Coll Cardiol..

[47] Yang J (2016). Targeting LOXL2 for cardiac interstitial fibrosis and heart failure treatment. Nat Commun..

[48] Binger T (2009). Migration potential and gene expression profile of human mesenchymal stem cells induced by CCL25. Exp Cell Res..

