# Supplementary material for: Mesenchymal stromal cells inhibit NLRP3 inflammasome activation in a model of Coxsackievirus B3-induced inflammatory cardiomyopathy
Source: Sci Rep. 2018 Feb 12;8:2820. doi: 10.1038/s41598-018-20686-6 (PMC5809634; doi:10.1038/s41598-018-20686-6)
Supplement: Supplementary file 1 — Supplementary Information [file 41598_2018_20686_MOESM1_ESM.doc]

**SUPPLEMENT**

**Mesenchymal stromal cells inhibit NLRP3 inflammasome activation in a model of Coxsackievirus B3-induced inflammatory cardiomyopathy**

Kapka Miteva^*,§^ PhD, Kathleen Pappritz^*,§^ PhD, Marzena Sosnowski*, Muhammad El-Shafeey^*^, Irene Müller^*,§^ PhD, Fengquan Dong^*^, Konstantinos Savvatis^*^, Jochen Ringe^*,#^ PhD, Carsten Tschöpe^*,†,§^ MD, Sophie Van Linthout^*,†,§^ PhD

* Berlin-Brandenburg Center for Regenerative Therapies, Charité, University Medicine Berlin, Campus Virchow, Berlin, Germany;

^†^ Charité-University-Medicine Berlin, Campus Rudolf Virchow, Department of Cardiology, Berlin;

^§^ DZHK (German Center for Cardiovascular Research), partner site Berlin, Germany;

#Laboratory for Tissue Engineering, Charité, University Medicine Berlin, Berlin, Germany

**Corresponding author:**

Sophie Van Linthout, PhD

Berlin-Brandenburg Center for Regenerative Therapies

Charité – University of Medicine Berlin

Campus Virchow

Südstrasse 2

13353 Berlin

e-mail: sophie.van-linthout@charite.de

Phone: +49-(0)30-450 539 486

Fax: +49-(0)30-450 539 409

**Supplemental Figures.**


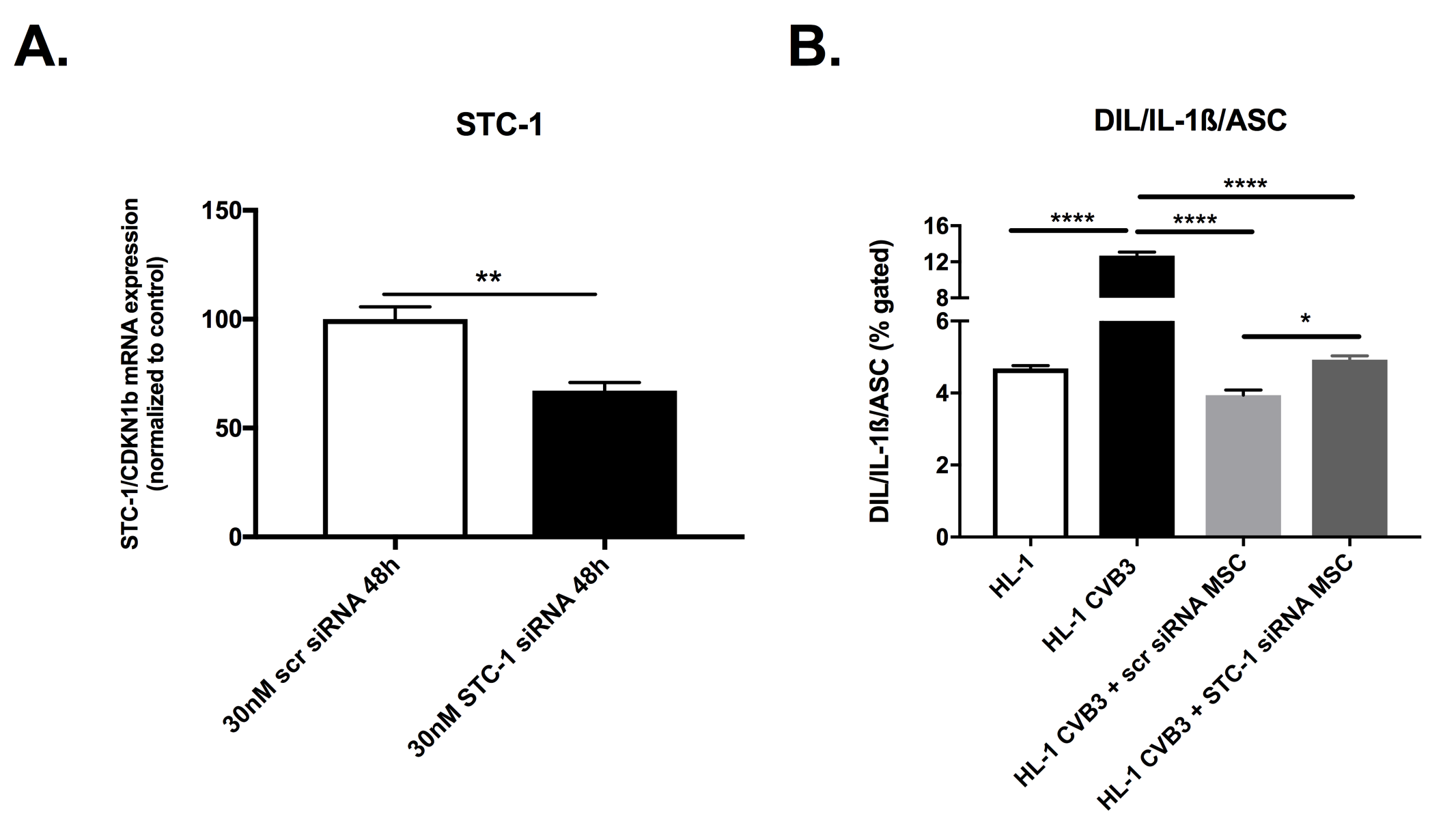


Supplemental Figure 1. The inhibitory effect of mesenchymal stromal cells on Coxsackievirus B3-induced NLRP3 inflammasome activity in HL-1 cells is partly abrogated by stanniocalcin knockdown. (*A*) Knockdown efficiency of stanniocalcin (STC)-1 in human MSC following transfection with 30 nM STC-1 siRNA and culture for 48h. Bar graphs represent the mean ± SEM of STC-1 expression in human MSC normalized to CDKN1b and depicted with MSC transfected with 30 nM scrambled (scr) siRNA set as 100% (white bar; n=6) and MSC transfected with 30 nM STC-1 siRNA (black bar; n=5), (*B*) Bar graphs represent the mean ± SEM of Dil^+^ IL-1ß^+^ ASC^+^ cells depicted as the percentage (%) gated, in control HL-1 cells (white bars; n=7), HL-1 cells infected with Coxsackievirus B3 (CVB3) (black bar; n=7), and CVB3-infected HL-1 co-cultured with MSC transfected with scr siRNA (light grey bar; n=4) or STC-1 siRNA (dark grey bar; n=5) with *p<0.05, **p<0.005, and ****p<0.0001.

**
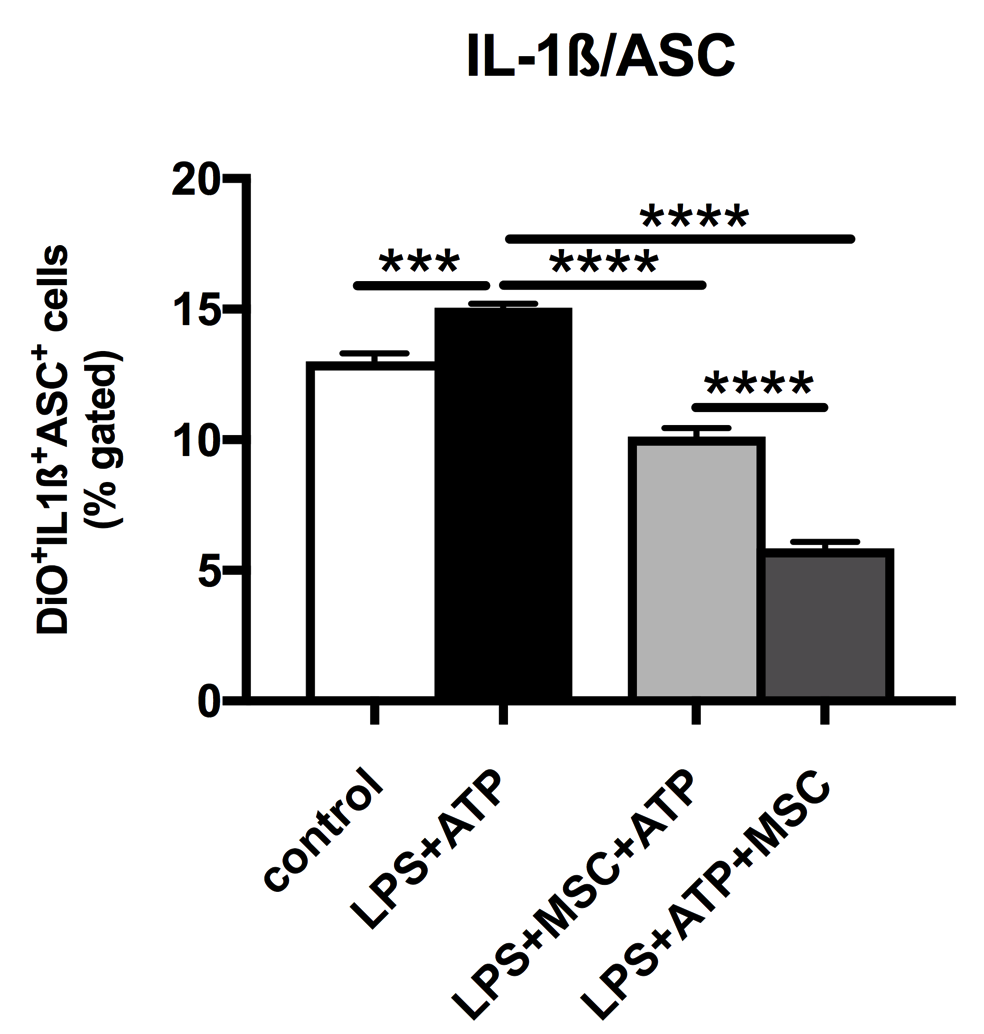
**

Supplemental Figure 2. Mesenchymal stromal cells suppress NLRP3 inflammasome activation upon classical activation with lipopolysaccharide and adenosine-triphosphate in HL-1 cells. Bar graphs represent the mean ± SEM of DiO^+^ IL-1ß^+^ ASC^+^ cells depicted as the percentage (%) gated, in control HL-1 cells (white bars; n=6), HL-1 cells supplemented with lipopolysaccharide (LPS) and adenosine-triphosphate (ATP) (black bar; n=8), and HL-1 supplemented with LPS and ATP with MSC added at the moment of LPS (light grey bar; n=10) or at the moment of ATP supplementation (dark grey bar; n=9) with ***p<0.0005, ****p<0.0001.

**
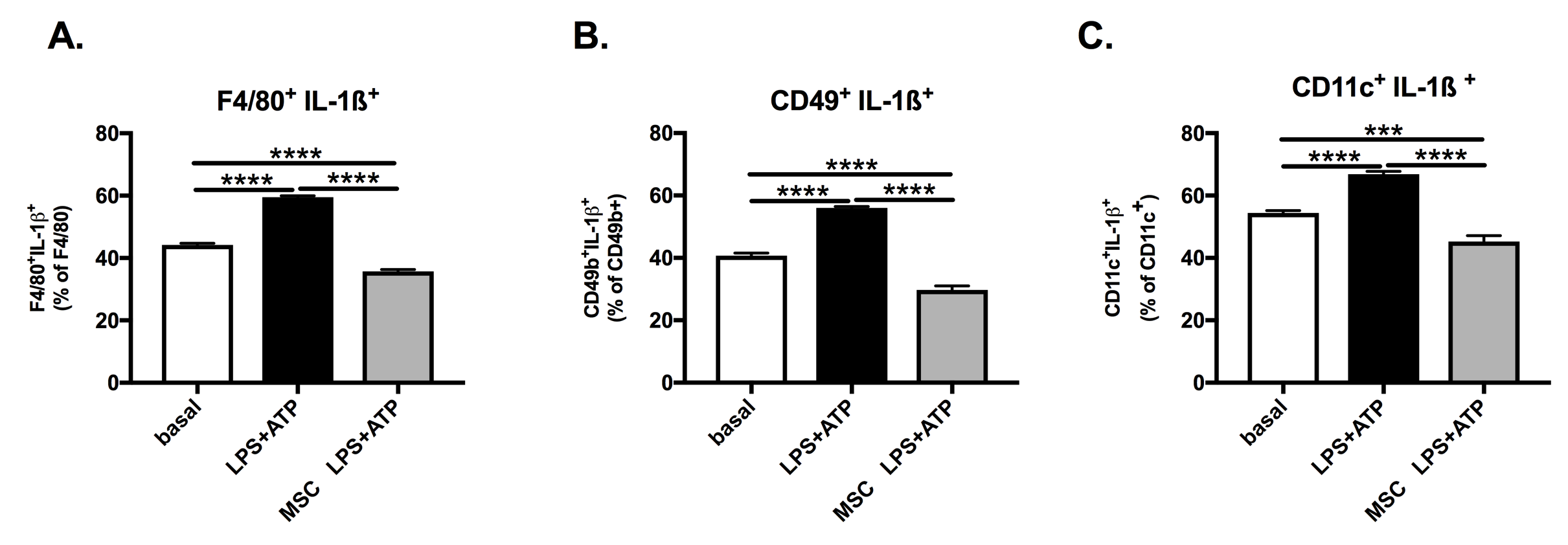
**

**Supplemental Figure 3. Mesenchymal stromal cells suppress NLRP3 inflammasome activation upon classical activation with lipopolysaccharide and adenosine-triphosphate in splenic macrophages, natural killer cells and dendritic cells.** Bar graphs represent the mean ± SEM of (*A*) F4/80^+^/IL-1β^+^, (*B*) CD49b^+^/IL-1β^+^, and (*C*) CD11c^+^/ IL-1β^+^ cells expressed as % F4/80^+^, CD49b^+^, and CD11c^+^ cells, respectively, in splenocytes under basal conditions (open bar; n=6), stimulated with lipopolysaccharide (LPS) and adenosine-triphosphate (ATP) (closed bar; n=6) and stimulated with LPS and ATP in the presence of MSC (grey bar; n=7), with ***p<0.0005, ****p<0.0001.

**Supplemental Figure 4. Correlation between NLRP3 and markers of Ca^2+^ homeostasis and fibrosis.** Panel A-F demonstrate linear regression curves of NLRP3 to (*A*) SERCA, (*B*) phospholamban, (*C*) col1a1, (*D*) col3a1, (*E*) LOX1, and (*F*) LOXL2 LV mRNA expression.
